# Triage of documents containing protein interactions affected by mutations using an NLP based machine learning approach

**DOI:** 10.1186/s12864-020-07185-7

**Published:** 2020-11-10

**Authors:** Jinchan Qu, Albert Steppi, Dongrui Zhong, Jie Hao, Jian Wang, Pei-Yau Lung, Tingting Zhao, Zhe He, Jinfeng Zhang

**Affiliations:** 1 Department of Statistics, Florida State University, Tallahassee, FL 32306 USA; 2 Laboratory of Systems Pharmacology at Harvard Medical School, Boston, MA 02115 USA; 3 CloudMedx, Palo Alto, CA 94301 USA; 4 Verisk – Insurance Solutions, Middletown, CT 06457 USA; 5 Department of Geography, Florida State University, Tallahassee, FL 32306 USA; 6 College of Communication and Information, Florida State University, Tallahassee, FL 32306 USA

**Keywords:** Protein-protein interactions, Mutations, Text mining, Biomedical literature retrieval, Protein interactions affected by mutations

## Abstract

**Background:**

Information on protein-protein interactions affected by mutations is very useful for understanding the biological effect of mutations and for developing treatments targeting the interactions. In this study, we developed a natural language processing (NLP) based machine learning approach for extracting such information from literature. Our aim is to identify journal abstracts or paragraphs in full-text articles that contain at least one occurrence of a protein-protein interaction (PPI) affected by a mutation.

**Results:**

Our system makes use of latest NLP methods with a large number of engineered features including some based on pre-trained word embedding. Our final model achieved satisfactory performance in the Document Triage Task of the BioCreative VI Precision Medicine Track with highest recall and comparable F1-score.

**Conclusions:**

The performance of our method indicates that it is ideally suited for being combined with manual annotations. Our machine learning framework and engineered features will also be very helpful for other researchers to further improve this and other related biological text mining tasks using either traditional machine learning or deep learning based methods.

## Background

Each cell of an organism contains a network of chemical reactions involving various types of molecules. The most important molecules within these networks are proteins which play a variety of roles within the cell: enzymes that catalyze chemical reactions, messengers to transmit signals to other cells, carriers of atoms and molecules within and between cells, and other roles some of which may still be unknown. Proteins rarely act alone, and they interact with one another or with other biomolecules in complex biological systems within the processes of life. There is a growing interest in seeking specific protein-protein interactions (PPIs) as drug targets, and though this presents a challenge previously considered insurmountable, there have already been some successes [1, 2]. Disruptions of PPIs by mutations can have severe impact on health, leading to cancer, degenerative diseases, and other serious illnesses. There is a rapidly growing body of literature documenting the mutations that can affect protein-protein interactions [3–6]. However, such information has been scattered in the literature as raw text.

A searchable database of information on protein-protein interactions affected by mutations (abbreviated as PPIAM in this paper) would be of great benefit to researchers for developing drugs targeting at PPIs, identifying novel biomarkers for a disease, and investigating other topics of potential relevance to precision medicine. Such information can also be very useful in automatic knowledge discovery [7] and integrative analysis of high-throughput genomics data [8–11]. However, reading and curating the information from literature manually to build the database would be very time and resource consuming. Computational methods to extract such information automatically or filter out irrelevant texts to assist manual annotations would be very helpful for building the database. The successful development of such systems could have an immediate impact to biomedical research.

A considerable number of computational methods have been developed to address a similar problem - extracting PPI information from literature [12–29]. These methods approached and addressed the problem using various techniques ranging from relatively simple co-occurrence, to rule-based pattern matching, to machine learning and deep learning based methods, which can be further enhanced by sophisticated natural language processing (NLP) techniques. However, these methods are not yet powerful enough to replace human experts in directly extracting protein-protein interactions from text. The added challenge of extracting PPIAM makes the problem substantially more difficult.

In 2004, BioCreative was initiated as an international, community-wide effort for evaluating text mining and information extraction methods applied to the biological and biochemical domains. BioCreative focuses on developing common standards and benchmark datasets for evaluating biological text mining systems. Friendly competitions are held among researchers developing systems to tackle well-defined problems. The systems are then evaluated based on gold standard datasets compiled and annotated by experts.

Triage for biological articles or abstracts has received considerable attention [30–39]. The problem of building models to triage abstracts containing at least one mention of a protein-protein interaction was addressed in the BioCreative II challenge in 2007 [40]. The most successful models employed SVM (support vector machine) classifiers with n-gram features and NLP preprocessing techniques such as stemming, part of speech tagging, and shallow parsing. This problem continued to be addressed in subsequent challenges, with a system developed by S. Kim and W.J. Wilbur for the BioCreative III challenge in 2011 forming the basis for the previously mentioned tool, PIE *the search* [41–43]. This system extracts gene names from articles, identifies MeSH terms, performs dependency parsing in addition to stemming and part of speech tagging, and feeds a collection of generated features to a support vector machine classifier with Huber loss. Since then, much work has been done on extracting protein-protein interactions from biological text, though no system is yet strong enough to outperform human curators.

The precision medicine track of the Biocreative VI challenge held in 2017 consisted of two tasks related to the automatic identification of descriptions of PPIAM in biomedical texts. For the first task, participants were asked to build systems capable of identifying if an abstract/paragraph contains at least one mention of a PPIAM. For the more challenging second task, participants were asked to develop systems capable of directly extracting mentions of PPIAM from biomedical texts. This study describes our work on the first task of precision medicine track of the BioCreative VI.

## Results

The competition results are shown in Table 1. The baseline model is the SVM classifier trained by the curators discussed in Methods Section. We submitted our results as team 433.

**Table 1:** Result of document triage task of the BioCreative VI precision medicine track. Our team ID is 433. Our method achieved highest recall and comparable F1 score among all the methods better than the baseline model

Team	Precision	Recall	F1	AvPr
421	0.6073	0.7997	0.6904	0.7253
418	0.6289	0.7656	0.6906	0.7185
374	0.6070	0.7898	0.6864	0.6929
375	0.5783	0.7713	0.6610	0.6822
433	0.5413	0.8835	0.6713	0.6632
Baseline	0.6122	0.6435	0.6274	0.6515
420	0.5438	0.8736	0.6703	0.6439
419	0.5992	0.6222	0.6105	0.6334
405	0.5484	0.5710	0.5595	0.5871
414	0.5022	0.9801	0.6641	0.5008
379	0.4649	0.3480	0.3981	0.4904

For the four other teams that outperformed the baseline model, Team 421 from the Dalian University of Technology employed a stacking ensemble of five individual neural network models, Team 418 from the National Technical University of Athens employed a sophisticated neural network with a reusable sequence encoder architecture, Team 374 from the University of Aveiro employed a deep learning approach with combinations of convolutional and LSTM networks, and only Team 375 from The University of Melbourne applied manual feature engineering and classical NLP techniques as what our team did. When evaluated by F1 score, Team 414 from Marmara University seems to have benefited from the balance of class labels in the test data. It is likely that they submitted a run consisting of almost all positive predictions [44].

After the competition, we fixed several bugs in our feature engineering pipeline and investigated the impact of adding features based on pre-trained word embeddings. It was our hypothesis that some of the superiority of the neural network based approaches could be explained by using information from the word embeddings, which is a necessary component of modern deep learning based NLP.

Post competition improvements are shown in Table 2. Curiously, adding word embeddings did not improve cross- validation results on the training set, but substantially improved the results on the test set. It seems that the word embeddings improved the model’s ability to generalize. This is reasonable since the pre-trained word embeddings contain information from a corpus, which is larger than the data used in this competition by several orders of magnitude.

**Table 2:** Post-competition improvement of our method. +w2v is the revised method with word2vec embedding

Model	Validation	Precision	Recall	F1	AvPr
Original	10f CV (Train)	0.6253	0.8208	0.7098	0.7148
Original	Test	0.5823	0.8096	0.6774	0.6785
+w2v	10f CV (Train)	0.6264	0.8150	0.7084	0.7138
+w2v	Test	0.5651	0.8509	0.6791	0.6962

Our final model’s average precision on the test set would have been good enough to achieve the 3rd place on the competition leaderboard, though a gap in the performance still remains between our model and the top two neural network based models.

**Fig. 1:**
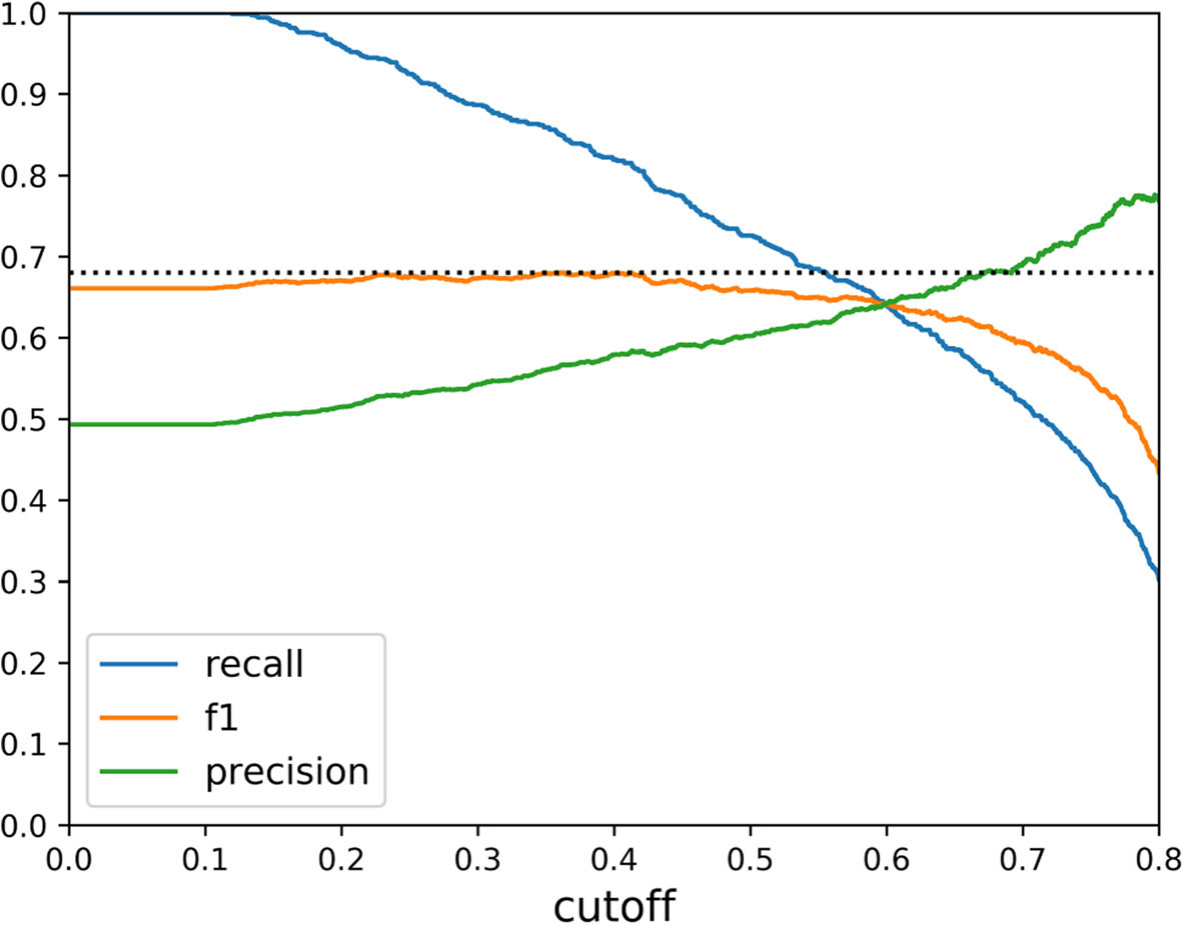
Plot of precision, recall and F1 score versus cutoff value

Figure 1 illustrates how the precision, recall, and F1 score depend on the choice of cutoff *p*. Our choice of 0.35 for the cutoff was fortunate, being close to the optimal value of 0.36. As pointed out in Method Section, the naïve model that classifies all abstracts as relevant is fairly competitive in terms of F1 score. This implies that in a situation where we expect roughly as many relevant abstracts as non-relevant ones, it is reasonable to manually review all abstracts. In real world situations where non-relevant abstracts vastly outnumber relevant ones, the triage systems developed for this competition would be more useful.

## Discussion

We developed a model for predicting whether an abstract contains at least one mention of a protein-protein interaction affected by a mutation (PPIAM) for Document Triage Task of the BioCreative VI Precision Medicine Track. This was done using classical NLP techniques without the use of modern deep learning approaches. Our final model achieved satisfactory performance with highest recall and comparable F1-score among those models better than the baseline.

Our method uses NLP techniques such as dependency parsing, TF-IDF word embedding, and XGBoost with manually engineered features. Deep learning models were used by some other teams as discussed in Result Section, but they are not better than our model in all metrics. Our method has the highest Recall, and comparable F1 score. We used a simpler model, which is more efficient in computation. XGBoost is generally more interpretable than deep learning models. In real world cases when high recall is desired or computation resource is limited, our method would show advantage over other methods. One real world use case is to assist manual annotation of documents, for which higher recall from the predictive model is usually desired. In such applications, one would group all instances of the same interaction together and annotate first those cases with highest predicted probabilities of being true. With this strategy, once an instance is validated, the other instances of the same interaction, for which a model has less confidences, do not need to be annotated. This will effectively improve the precision and overall F1 of the model being used.

## Conclusion

The performance of our NLP based machine learning model makes it an ideal method for being combined with manual annotations in extracting PPIAM from literature. Our machine learning framework and engineered features will be very helpful for other researchers to further improve their biological text mining tasks using either traditional machine learning or deep learning based methods.

## Methods

### Data and evaluation

Training and test datasets were constructed by five professional BioGRID PPI database [45] curators. For the training set, 2852 abstracts were drawn from the IntAct database which were already annotated for protein-protein interactions and any mutations influencing them [46]. Additional articles were selected using PIE *the search* [43], a text mining tool for protein interactions, to identify PubMed abstracts that likely contain protein-protein interactions. tmVar was then applied to identify mentions of mutations [47]. Roughly 1200 abstracts were selected and then manually reviewed and annotated by the curators. A total of 4082 PubMed abstracts were included in the training corpus with 1729 of them labeled as containing at least one PPIAM (positive) and 2353 of them labeled as negative. No previously annotated articles were included in the test corpus. All abstracts were selected using text mining tools and manually reviewed by the curators. The test corpus consisted of 1427 abstracts with 704 being assigned a positive label and 723 being assigned a negative label. In the following, we will call abstracts assigned a positive label relevant and abstracts assigned a negative label not relevant or irrelevant. More details on the procedures used to build these corpora can be found in the BioCreative VI precision medicine track paper [48].

The participating teams were given the annotated training dataset and tasked with building models to identify whether a PubMed abstract mentions at least one PPIAM using information from the title and body text. Participating teams were encouraged to build models that can generate confidence scores by estimating the probability that a given paragraph of text contains a PPIAM. An unlabeled copy of the test corpus was provided to each participating team near the end of the competition and teams submitted the predicted labels and confidence scores computed by applying their models on this corpus. Teams were permitted to submit three sets of predictions. The model was trained through 10-fold cross-validation on the training data for hyperparameter tuning. We then fit the model using all the training data and making predictions on the test data. Several standard metrics applicable to information retrieval problems including precision, recall, F1 score, and average precision were used. The curators constructed a baseline linear support vector machine (SVM) classifier that made use of unigram and bigram features [49, 50].

Precision, recall, and F1 score are all based on comparing the true labels to the predicted labels in the evaluation set, while average precision is based upon comparing the true labels to the numerical confidence scores. Precision is given by the number of true positives divided by the number of abstracts predicted to be relevant by the model. It measures the quality of positive predictions. Recall is defined as dividing the number of true positives by the number of relevant abstracts in the evaluation set. It measures the sensitivity of the model.

$$ \mathrm{precision}=\frac{\mathrm{true}\ \mathrm{positives}}{\mathrm{true}\ \mathrm{positives}+\mathrm{false}\ \mathrm{positives}} $$

$$ \mathrm{recall}=\frac{\mathrm{true}\ \mathrm{positives}}{\mathrm{true}\ \mathrm{positives}+\mathrm{false}\ \mathrm{negatives}} $$

F1 score is given by the harmonic mean of precision and recall, which aims to balance the tradeoff between the two metrics.

$$ {F}_1=\frac{2}{\frac{1}{\mathrm{precision}}+\frac{1}{\mathrm{recall}}} $$

Given a set of confidence scores predicted on an evaluation set, one can compute predicted labels in multiple ways by choosing different probability cutoff, *p*. All abstracts with predicted probability greater than *p* are predicted as relevant and others are predicted as not relevant. Average precision (AvPr) is a cutoff-independent metric given by the area under the precision-recall curve [51]. It is similar to the ROC-AUC (the area under the precision-recall curve) metric, and is often used for information retrieval because it is considered to be a more informative metric for data with imbalanced class distribution [52].

In real applications, a large imbalance in class distribution should exist for this triage problem: there should be much fewer relevant abstracts than non-relevant ones. In the corpora used in the competition, this is not the case. Having balanced class distribution in the test corpus has implications for the metrics used in the competition. A model that simply classifies all abstracts as relevant has a better F1 score than the baseline SVM model [53]. However, when judging by average precision, the SVM model is clearly superior. None of the models submitted in the competition achieved an F1 score dramatically larger than the “naïve” all relevant classifier.

The baseline results show that there are difficulties in generalizing from the IntAct data to the data manually reviewed by the curators. This can be seen in Table 3, which is taken from the precision medicine track corpus paper [53]. Training and evaluating on abstracts drawn from IntAct gives much better performance compared to training on the IntAct abstracts and evaluating on the remaining abstracts. For our own model, we observed that cross-validation on the dataset featuring a mixture of IntAct and manually reviewed abstracts yielded better results than those seen by training on IntAct and validating on the test data. We found that this difficulty in generalization was somewhat mitigated by the inclusion of features from pre-trained word embeddings.

**Table 3:** Baseline model performance on the BioCreative VI precision medicine track corpus

Data	Precision	Recall	F1	F1 all relevant	AvPr
10f CV (IntAct)	0.7184	0.6321	0.6725	0.5507	0.7577
Validation (TM)	0.6210	0.6897	0.6536	0.6842	0.6551
10f CV (all data)	0.6891	0.6260	0.6561	0.5915	0.7225

*AvPr:* Average precision; *10f CV:* 10-fold Cross-validation; *TM:* Text Mining set, corpus of abstracts found with the aid of text mining methods

### Modeling process

**Fig. 2:**
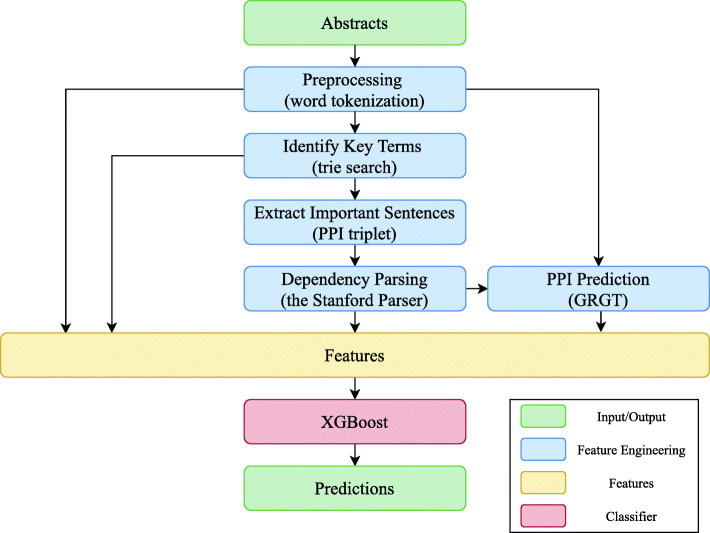
Illustration of our method. In the “Feature Engineering” boxes, the major tools/algorithms used in each step are mentioned in the parentheses

In an initial preprocessing step, the abstracts were tokenized into sentences and words. Then key terms were identified, and important sentences were then extracted based on the presence of the key terms. These sentences were then dependency parsed. A protein-protein extraction system previously developed was then applied based on features extracted during the previous steps [28, 54, 55]. Finally, features were extracted from preceding steps in the pipeline and were used as input for training an XGBoost classifier [56]. A broad outline of the system is illustrated in Fig. 2. We now explain each of the steps in more detail.

#### Preprocessing

The NLTK python package was used for tokenization [57]. Sentence tokenization was accomplished using NLTK’s implementation of the Punkt tokenizer custom trained on all abstracts in the training data [58]. This was done because the default sentence tokenizer had difficulty in identifying abbreviations not commonly found in ordinary English text. Word tokenization was performed with NLTK’s default method.

#### Identification of key terms

Protein mentions, interaction words, and mutation-related words were identified with a simple keyword search. A list of protein names was taken from the UniProt Swiss-Prot database [59]. We implemented a text search algorithm to identify protein mentions based on a simple trie search [60]. Appositive statements such as *“Tubulin folding cofactor A (TFCA), which captures …*” were simplified by removing the duplicate mention (e.g., an acronym after its full name). This instance would be changed to *“Tubulin folding cofactor A, which captures …”* .

A dictionary of interaction words was taken from a previous study. It was constructed based on the personal knowledge of the authors and manual review of sentences in the literature known to contain protein-protein interactions [21]. This dictionary has been successfully employed in similar applications [7, 28, 61–64].

We compiled a dictionary of mutation-related words for this challenge using a combination of manual and computational approach. For each term *t*, we calculated its frequency *f*_+_(*t*) for the abstracts with the positive label and compared it to the frequency *f*_−_(*t*) for the abstracts with the negative label. Terms with a large value of ∣*f*_+_(*t*) − *f*_−_(*t*)∣ were manually reviewed and mutation related words were identified. During validation, terms not included in a training fold were excluded when making predictions on the corresponding test fold. This was done to avoid a potential information leak. In addition, terms based on the standard mutation nomenclature, such as R117H, were identified using regular expressions and included as mutation-related words [65].

#### Extraction of important sentences

Important sentences were identified for further attention. These included sentences containing at least two protein mentions and an interaction word, and sentences containing at least one protein mention and one mutation related word. Two protein mentions and an interaction word in the same sentence are referred to as a protein-protein interaction (PPI) triplet. A protein mention and a mutation related word in the same sentence are referred to as a protein-mutation pair.

#### Dependency parsing

**Fig. 3:**
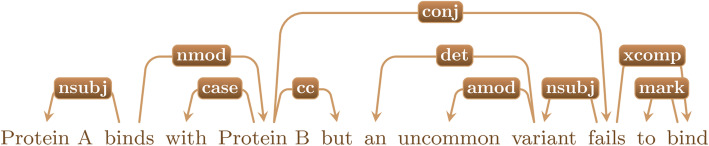
An example of dependency parsing. The labeled arcs describe the dependency between two words

Important sentences were then parsed using the Stanford Fast Neural Network based Dependency Parser [66]. Given a sentence, the parser constructs a directed graph with terms from the sentence as nodes and grammatical relations between the terms as edges. To simplify the parsing, protein names were replaced with the single term identifiers such as PROT1, or PROT2. Figure 3 gives an example of a parsed sentence.

#### PPI prediction

A model called GRGT (Grammatical Relationship Graph for Triplets), which was previously developed was then used to predict the probabilities of each PPI triplet in an abstract being a true protein-protein interaction [67]. This model extracts features from the shortest paths between the key terms in a PPI triplet and from semantic patterns proposed in an earlier work [21].

#### Features

Feature extraction was accomplished using the popular Python library scikit-learn [68].

##### Unigrams and bigrams

We began with classic unigram and bigram features. For these, tokens were stemmed and stop words were removed. To reduce the dimension of the feature space, only the most frequent unigrams and bigrams in the training corpus were included, with the number of unigrams and bigrams to use determined through cross-validation. Unigrams and bigrams were TF-IDF vectorized, with the value for each unigram or bigram feature in an abstract equal to the term frequency-inverse document frequency of the term in that abstract [69]. TF-IDF gives a measure of the importance of a term within a document. If *t* is a term and *d* a document from a family of documents *D*, then

$$ \mathrm{TF}-\mathrm{IDF}\left(t,d,D\right)=\mathrm{TF}\left(t,d\right)\mathrm{IDF}\left(t,D\right) $$

where

$$ \mathrm{IDF}\left(t,D\right)=\log \frac{N}{\left\Vert \left\{d\in D:t\in d\right\}\right\Vert } $$

with *N* being the total number of documents in the dataset and ‖{*d* ∈ *D* : *t* ∈ *d*}‖ being the number of documents in the dataset that contain term *t*. A term is more important to a document if it occurs many times in that document, but its importance is penalized if it occurs frequently in the entire dataset.

##### Frequencies of key terms

Three additional features were the numbers of protein mentions, interaction words, and mutation words in an abstract divided by the total number of tokens in the abstract. These are shallow features that would not distinguish between the sentences “PROT1 interacts with PROT2” and “PROT1 does not interact with PROT2”. Their utility derives from statistical properties of the corpus rather than any information about the meaning of the texts.

##### Shortest path counts in dependency parses

**Fig. 4:**
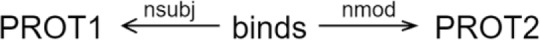
The shortest path between PROT1 and PROT2 in the example shown in Fig. 3 (they are shown as “Protein A” and “Protein B” in Fig. 3)

The shortest path between two terms in a dependency graph tends to contain the important information describing their relation [70]. Consider for instance the shortest (undirected) path between PROT1 and PROT2 in Fig. 4.

It is reasonable that the length of the shortest path between two terms gives information about the strength of their relationship.

For each PPI triplet, we found the shortest path between the two protein mentions and the shortest paths between each protein mention and the corresponding interaction word. For each protein-mutation pair, we found the shortest path between the protein mention and the mutation word.

Consider two terms, *w*_1_ and *w*_2_, within a sentence *S*. Let sp(*w*_1_, *w*_2_) denote the shortest path between *w*_1_ and *w*_2_, and Lsp(*w*_1_, *w*_2_) denote the length of the shortest path between *w*_1_ and *w*_2_. Given a PPI triplet (*p*_1_, *p*_2_, *iw*) within a sentence *S*, where *p*_1_ is the first protein mention to appear in *S*, *p*_2_ the second protein mention to appear, and *iw* the interaction word. We call sp(*p*_1_, *p*_2_), sp(*p*_1_, *iw*), and sp(*p*_2_, *iw*) shortest paths of the first, second, and third types, respectively. The path lengths Lsp(*p*_1_, *p*_2_), Lsp(*p*_1_, *iw*), and Lsp(*p*_2_, *iw*) were then computed. These path lengths could be features at the triplet level, but there may be many triplets within the same abstract. To generate features at the abstract level, we employed a binning procedure. We construct bins for shortest path lengths 1, 2, 3, 4, …, 10, and 11^+^. For each shortest path type, we counted the number of shortest paths within an abstract that fall into each bin. This gives a total of 33 additional features. The same procedure is employed for shortest paths sp(*p*, *mu*), between the protein *p* and mutation word *mu* in protein-mutation pairs.

#### PPI triplet predictions

For each PPI triplet in an abstract, we used the previously mentioned model to predict the probability of it being a true protein-protein interaction. We employed a similar binning procedure to generate abstract level features from these predictions. Based on an examination of the histogram of predicted probabilities for all triplets in all the training set, we constructed the probability bins [0, 0.3), [0.3, 0.5), [0.5, 0.7), and [0.7, 1.0]. For each abstract, counts of the number of triplets with predicted probability *p* falling into each bin were incorporated as features.

#### Additional shortest path features

##### Unigrams and bigrams along shortest paths

**Fig. 5:**
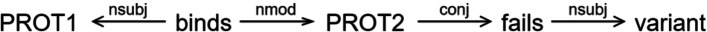
The shortest path between PROT1 and variant in the example shown in Fig. 3

As mentioned earlier, for each protein-mutation pair in an abstract we generated a dependency parse for its containing sentence and computed the shortest path in the dependency graph between the protein mention and the mutation-related word. Unigrams and bigrams along these shortest paths were included as features, with the direction of the path taken from the protein to the mutation related word. For example, Fig. 5 shows the shortest path between PROT1 and variant.

The unigrams along this path are binds, PROT2, and fails. The bigrams are binds/PROT2 and PROT2/fails. Abstract level features are then given by the unigrams and bigrams along the shortest paths in all protein-mutation pairs in an abstract. As before, these unigrams and bigrams were TF-IDF vectorized. Stop words were not removed. We believe that they may carry important information within a shortest path.

##### Unigrams and bigrams of dependency relations

Unigrams and bigrams of dependency relations along these shortest paths are also included. In the example shown in Fig. 5, the unigrams are **nsubj**, **nmod**, and **conj**, with **nsubj** appearing twice. These were vectorized by count instead of TF-IDF. Since most dependency relations appear in a large majority of abstracts, the TF-IDF of almost all dependency relation terms is equal to or very near zero.

#### Frequencies of key terms

Frequencies of key terms along the shortest paths were also included as features. We used the number of protein mentions in all shortest paths divided by the total number of terms in all shortest paths, and similarly for interaction words and mutation related words. Note that we do not include the protein mention at the start nor the mutation word at the end as members of a shortest path.

#### Incorporating PPI probabilities

The terms in the shortest path between a protein and mutation word should contain information that may help decide if the mutation affects the protein. However, it may not contain information about whether the abstract describes an interaction containing that particular protein. Information describing an interaction may be in a different sentence. We attempted to capture such information with the following scheme.

Given a protein-mutation pair (Prot, mu) in an abstract $\mathcal{A}$, we identified all PPI triplets in $\mathcal{A}$ that contain the protein Prot and found the highest probability *p* predicted by our PPI extraction algorithm among all such triplets. If *p* > 0.5, we call (Prot, mu) a positive pair, otherwise we call (Prot, mu) a negative pair.

All terms within the shortest paths corresponding to positive pairs then had a nonsense string appended, and the terms in the shortest paths corresponding to negative pairs had a different nonsense string attached so that they could be distinguished. Unigram and bigram features were then extracted for these augmented terms. The same procedure was also carried out for unigrams and bigrams of dependency relations. A new collection of shortest path features was also calculated. It consists of counts of positive pairs with path lengths in the bins 1, 2, 3, 4, …, 11+ and counts of negative pairs with path lengths in the bins 1, 2, 3, 4, …, 11 + .

#### Word embeddings

Significant improvement was seen when incorporating features based on pre-trained word embeddings. These were added after the competition. Word embeddings are generally dense, relatively low dimensional vector representations of words. We used a collection of word embeddings trained by Pysallo et al. on what was at the time a complete collection of PubMed abstracts and PubMed Central articles [71]. These were trained using the popular word2vec framework [72]. We built abstract level features from the word embeddings by taking the TF-IDF weighted average of all word embeddings in an abstract after removing the stop words. We also used the ordinary average of all word embeddings along the shortest paths from all protein-mutation word pairs within an abstract as features.

#### Models

In our initial experiments, we used the scikit-learn implementations of the random forest and linear SVM classifiers as baseline models [73, 74]. These were chosen because their performance is not as sensitive to the choice of hyperparameters as other commonly used models. Random forest models are typically sufficient to grow trees to maximum depth and ensemble as many trees as possible. Decision tree methods have the added benefit of not being sensitive to the scale of the features [75]. The linear SVM has a single regularization parameter that determines the penalty given for misclassification. We found that the effect of this parameter was consistent across different feature sets and set it to the default value in our experiments.

In our final model, we used a regularized gradient boosted trees classifier from the popular XGBoost library [56]. This is a powerful “off-the-shelf” model that has been successful in many data science competition. Hyperparameters were tuned using a grid search [76]. At the time of the competition, hyperparameters were chosen to optimize the ROC-AUC metric. For all the work done after the competition, this was changed to optimize the average precision.

Since gradient boosted trees do not tend to give accurate probability estimates, we used Platt scaling to calibrate these values to accurate probabilities [76]. This involved fitting a logistic regression to predict the class labels from the probabilities predicted by the gradient boosted trees model. This works because logistic regression predicts accurate probabilities by design. This probability calibration did not affect the competition metrics but was useful for interpreting our system’s output. An optimal cutoff value of 0.35 for the predicted probability *p* was chosen through cross-validation.

## Data Availability

The datasets used in our study are available in the BioCreative VI Track 4: Mining protein interactions and mutations for precision medicine repository, https://biocreative.bioinformatics.udel.edu/tasks/biocreative-vi/track-4/.

## Abbreviations

PPI: Protein-protein interaction
PPIAM: Protein-protein interactions affected by mutations
NLP: Natural language processing
SVM: Support vector machine
AVPR: Average precision
10f CV: 10-fold cross-validation
